# Supplementary material for: Intensiometric biosensors visualize the activity of multiple small GTPases in vivo
Source: Nat Commun. 2019 Jan 14;10:211. doi: 10.1038/s41467-018-08217-3 (PMC6331645; doi:10.1038/s41467-018-08217-3)
Supplement: Supplementary file 1 — Supplementary Information [file 41467_2018_8217_MOESM1_ESM.pdf]

Supplementary Information

Intensiometric biosensors visualize the activity of multiple small GTPases *in vivo*

Kim et al

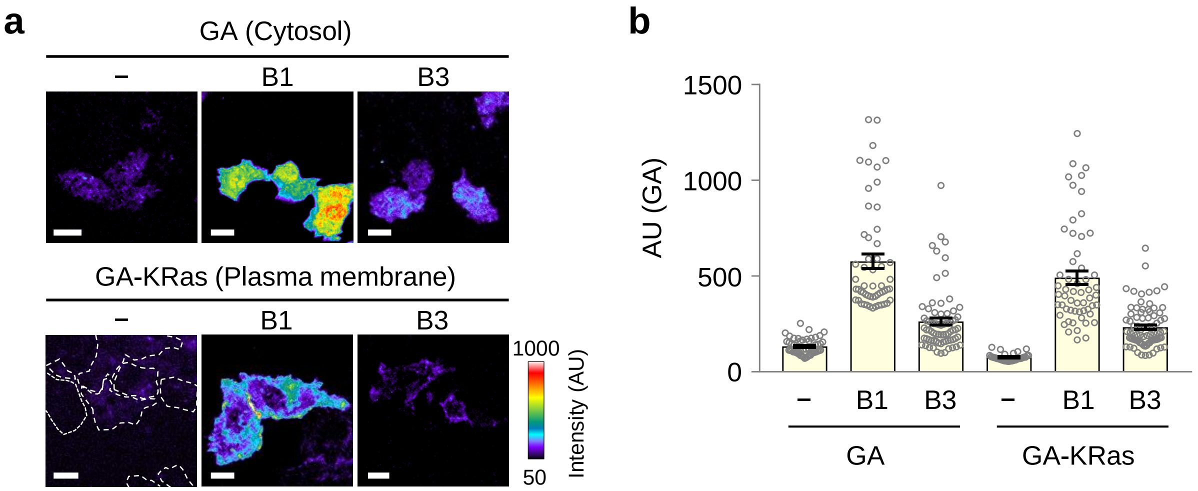

Supplementary Figure 1. Basal fluorescence generated by ddFP heterodimerization.
(a) Fluorescence images of HeLa cells co-expressing proteins as indicated. White dotted lines indicate cell boundaries. **(b)** Graph showing intensities of GA in HeLa cells for each combination. n = 53, 55, 74 (GA), 34, 56, 83 (GA-KRas). AU: arbitrary unit; Error bars, s.e.m. Scale bars, 20 µm.

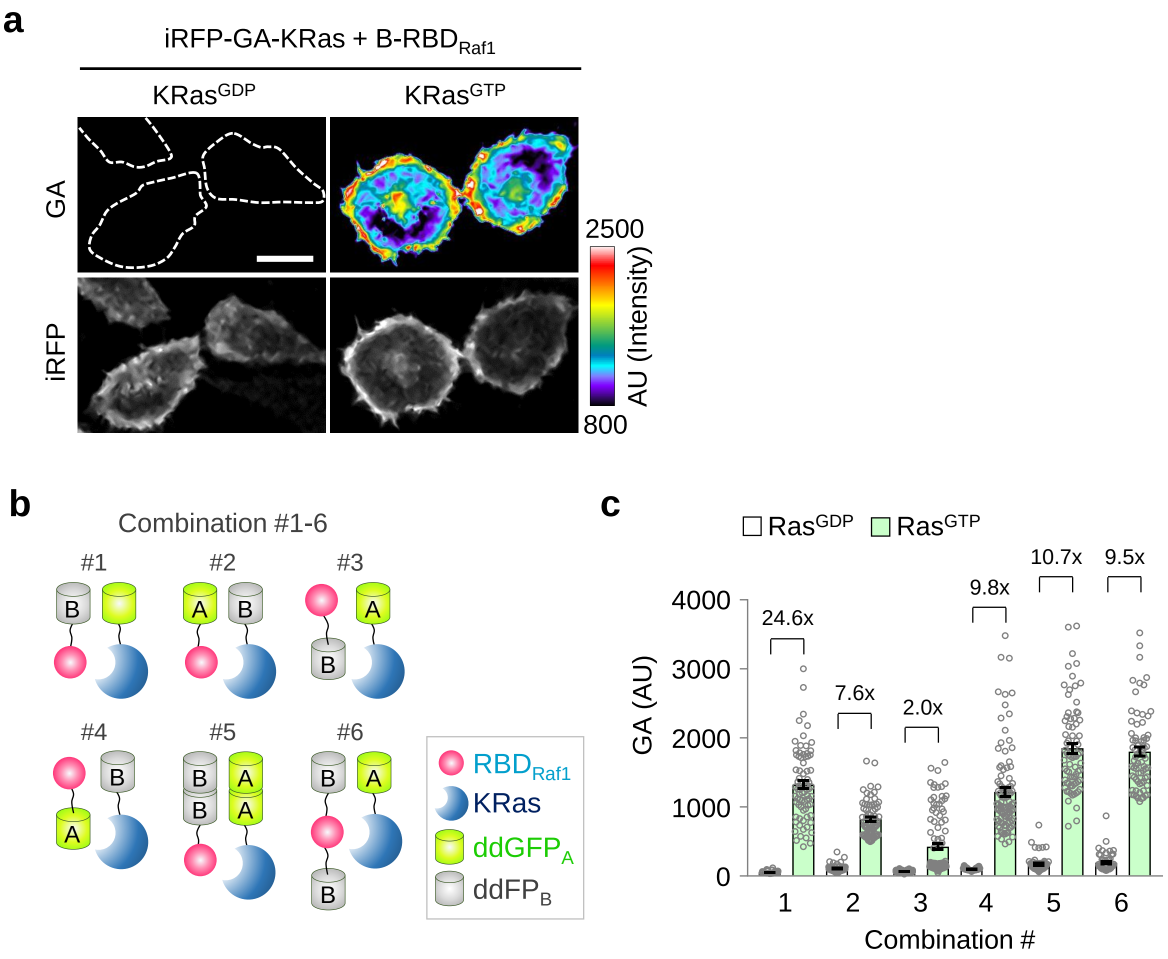

Supplementary Figure 2. Design and optimization of KRas sensor using ddFP-fused Ras and RBD_{Raf1}.

(a) Fluorescence images of HeLa cells co-expressing either iRFP-labeled GA-$KRas^{GTP}$ or GA-$KRas^{GDP}$ with B-RBD_{Raf1}. Images of GA and iRFP were displayed by pseudocolor and monochrome, respectively. The color bar indicates range of GA intensity. **(b)** Design of ddFP-based KRas sensor constructs. **(c)** Graph showing fluorescence intensity of GA for each set presented in **b**. In each set, either active (Ras^{GTP}) or inactive (Ras^{GDP}) was used. n = 77, 72 (Combination #1), 72, 111 (#2), 76, 97 (#3), 62, 66 (#4), 71, 53 (#5), 53, 52 (#6). AU: arbitrary unit; Error bars, s.e.m.

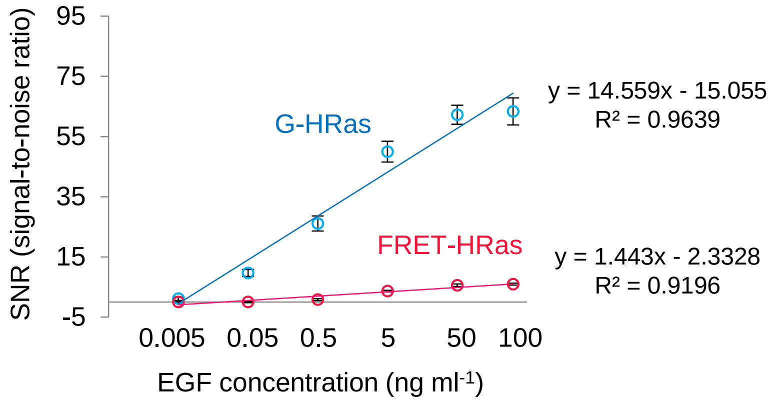

Supplementary Figure 3. Comparison of SNR (signal-to-noise ratio) between intensiometric and ratiometric Ras sensor.

Graph showing SNR (signal-to-noise ratio) quantification for both ddFP-based G-HRas and FRET-based RaichuEV-HRas under dose-dependent EGF treatment. *n* = 31, 37, 49, 31, 31, 33 for G-HRas and 29, 23, 25, 22, 43, 42 for RaichuEV-HRas. Error bars, s.e.m. See **Methods** for **SNR analysis** in detail.

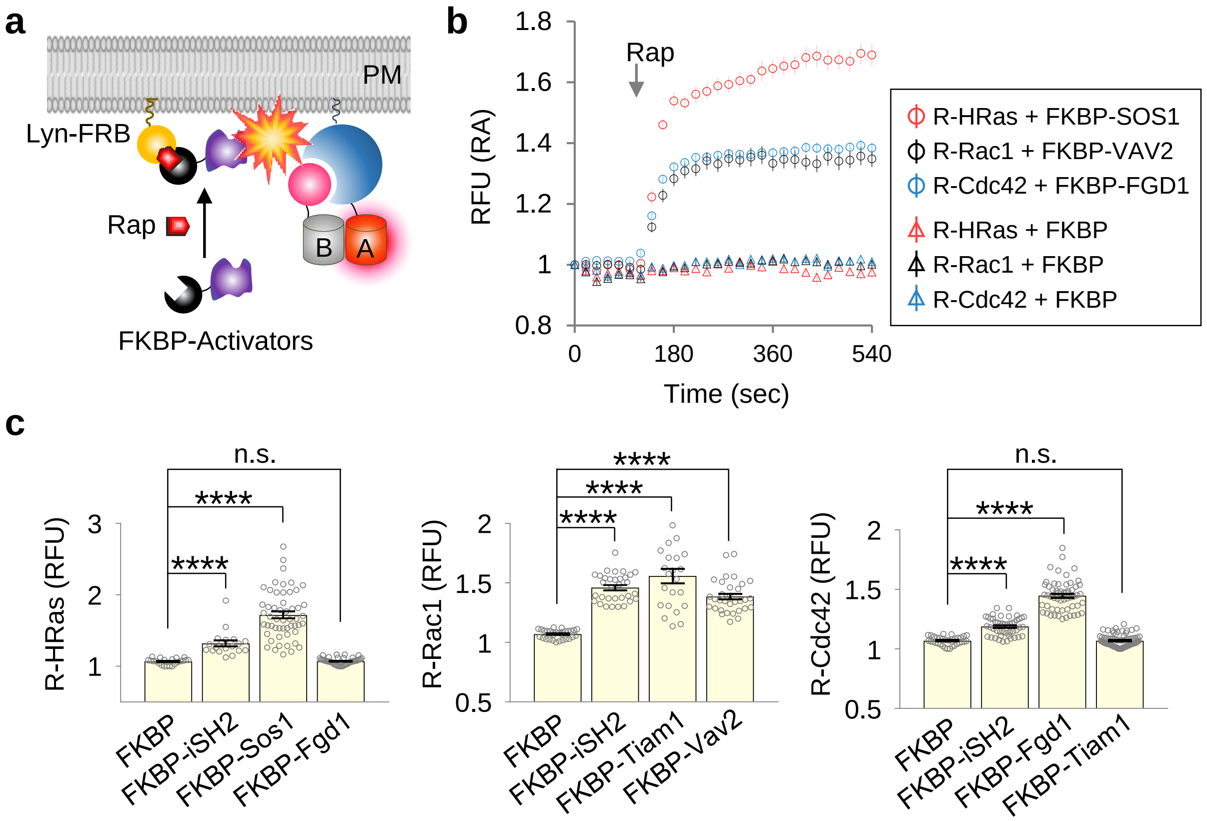

Supplementary Figure 4. Monitoring activities of various small GTPases under inducible activation of specific upstream signaling.

(a) Schematic showing rapamycin (Rap)-inducible recruitment of protein for small GTPase activation. **(b)** Time-lapse graph showing relative fluorescence changes of small GTPase sensors upon treatment of rapamycin at t = 120 s. Images were captured at 20-s intervals. n = 30, 22 (red circle, red triangle), 24, 30 (black circle, black triangle), 56, 33 (blue circle, blue triangle). Error bars, s.e.m. **(c)** Graphs showing maximal fold-changes of sensor signal upon recruitment of each activator. n = 22, 19, 53, 44 (left), 30, 32, 23, 32 (middle), 33, 49, 56, 77 (right). One way analysis of variance (ANOVA), P = 0.0012 for (FKBP only vs. FKBP-iSH2); $P < 0.0001$ for (FKBP only vs. FKBP-Sos1); $P = 0.9988$ for (FKBP only vs. FKBP-Fgd1); $P < 0.0001$ for (FKBP only vs. FKBP-iSH2, FKBP-Tiam1 or FKBP-Vav2); $P < 0.0001$ for (FKBP only vs. FKBP-iSH2, FKBP-Fgd1, FKBP-Tiam1). RFU: relative fluorescence unit; Error bars, s.e.m.; n.s., not significant.

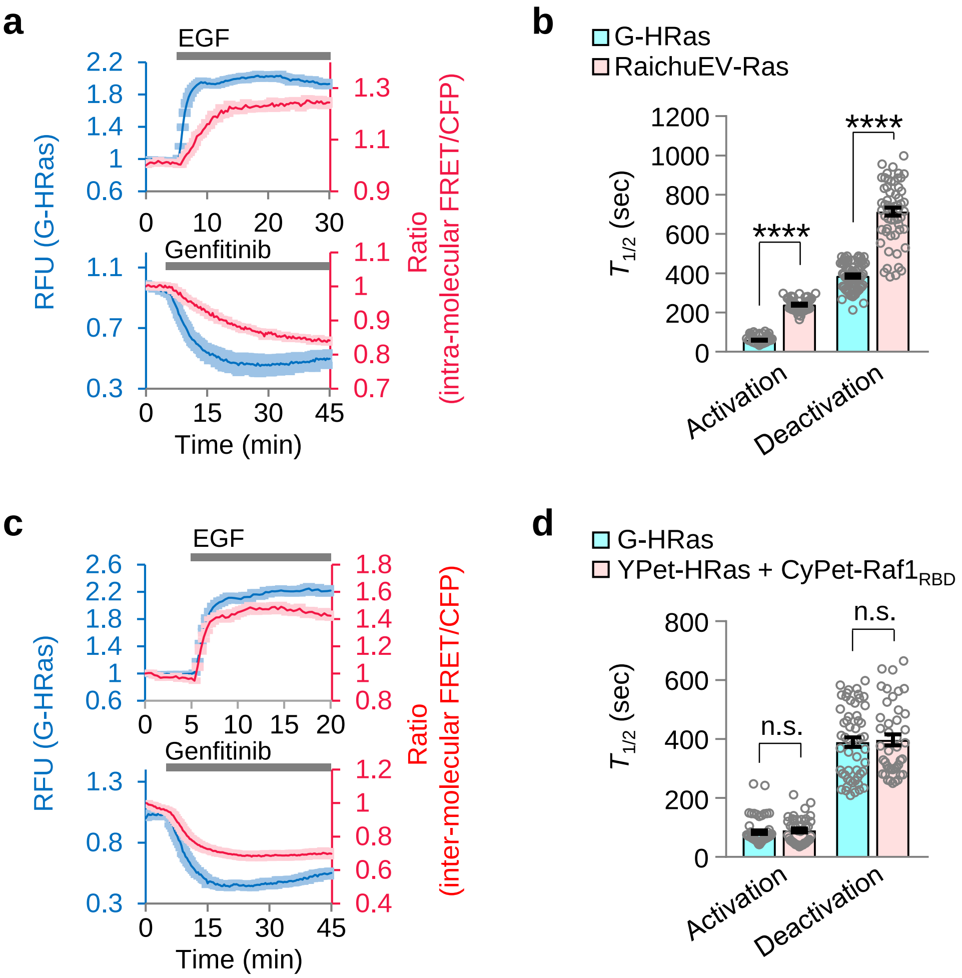

Supplementary Figure 5. Kinetic comparison of G-HRas sensor with Ras FRET sensor.

(a) Time-course measurement of ddFP-based and intramolecular FRET-based Ras sensor signals under activation or inhibition of EGFR signaling. **(b)** Graph showing Ras activation and deactivation kinetics represented by both sensors (n = 58, 46, 67, 58; **** $P < 0.0001$, **** $P < 0.0001$ by Student's two-tailed t-test; Error bars, s.e.m.). **(c)** Time-course measurement of ddFP-based and intermolecular FRET-based Ras sensor signals under activation or inhibition of EGFR signaling. **(d)** Graph showing Ras activation and deactivation kinetics represented by both sensors (n = 57, 41, 54, 42). RFU: relative fluorescence unit; Error bars, s.e.m.; n.s., not significant.

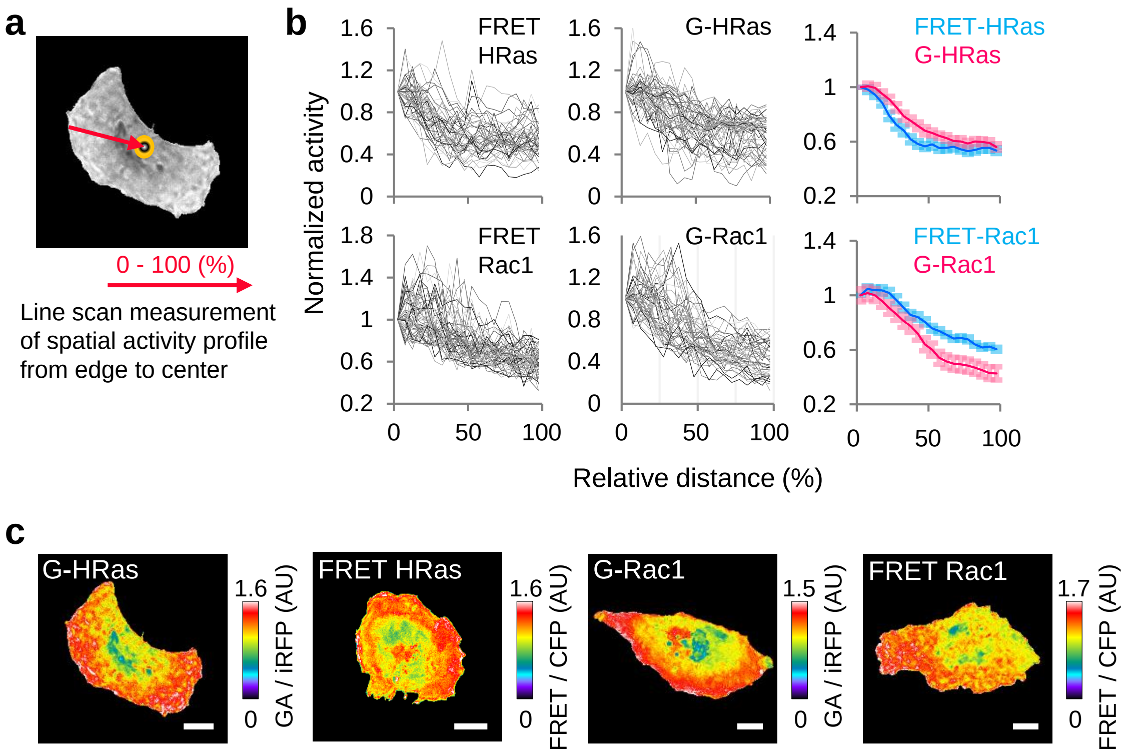

Supplementary Figure 6. Spatial distribution of small GTPase activity monitored by FRET- and ddFP-based sensors.

(a) Arrow on the monochrome image indicates the region of line scan to spatially profile small GTPase activity from protruding region to the center of the cell. The width of line was fixed for all analyzed cells and the line length was normalized in percentile. **(b)** Graphs showing relative changes of sensor activity as a function of distance from the cell edge. Mean values of sensor activity across cell regions are shown as red and blue lines. Error bars, s.e.m. **(c)** Fluorescence images representing spatial distribution of each protein activity during random migration of MDA-MB-231 cells. AU: arbitrary unit.

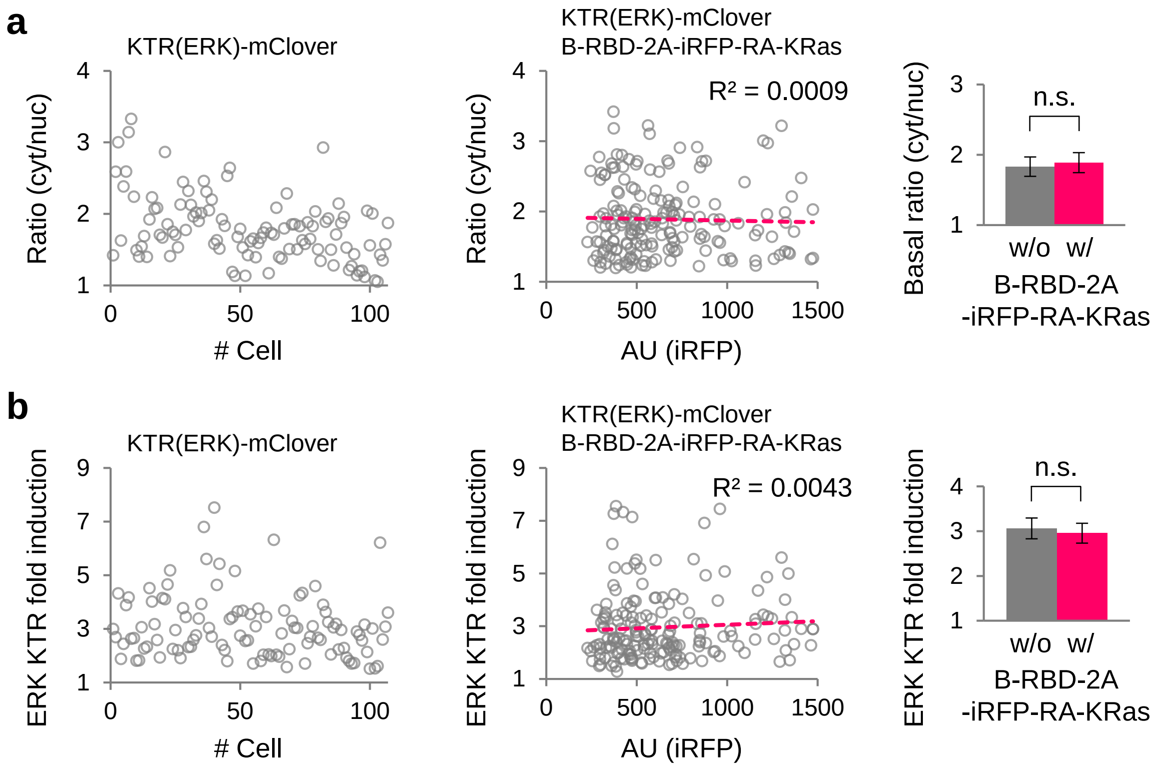

Supplementary Figure 7. Effect of R-KRas sensor expression on downstream signaling.
(a) Scatter plots showing distribution of basal ERK activity in individual cells measured by KTR(ERK) sensor without (left) or with (middle) iRFP-labeled R-KRas sensor. iRFP signals were used to estimate relative expression levels of R-KRas sensor. Each circle represents a single cell. *n* = 174, 177. (right) Average values of basal ERK activity for both groups. **(b)** Scatter plots showing distribution of maximal fold changes of ERK activity measured by KTR(ERK) sensor without (left) or with (middle) iRFP-labeled R-KRas sensor. (right) Average values of maximal fold changes of ERK activity for both groups. Transfected HeLa cells analyzed in **a** were stimulated by EGF (50 ng ml^{-1}). AU: arbitrary unit; Error bars, s.e.m.; n.s., not significant.

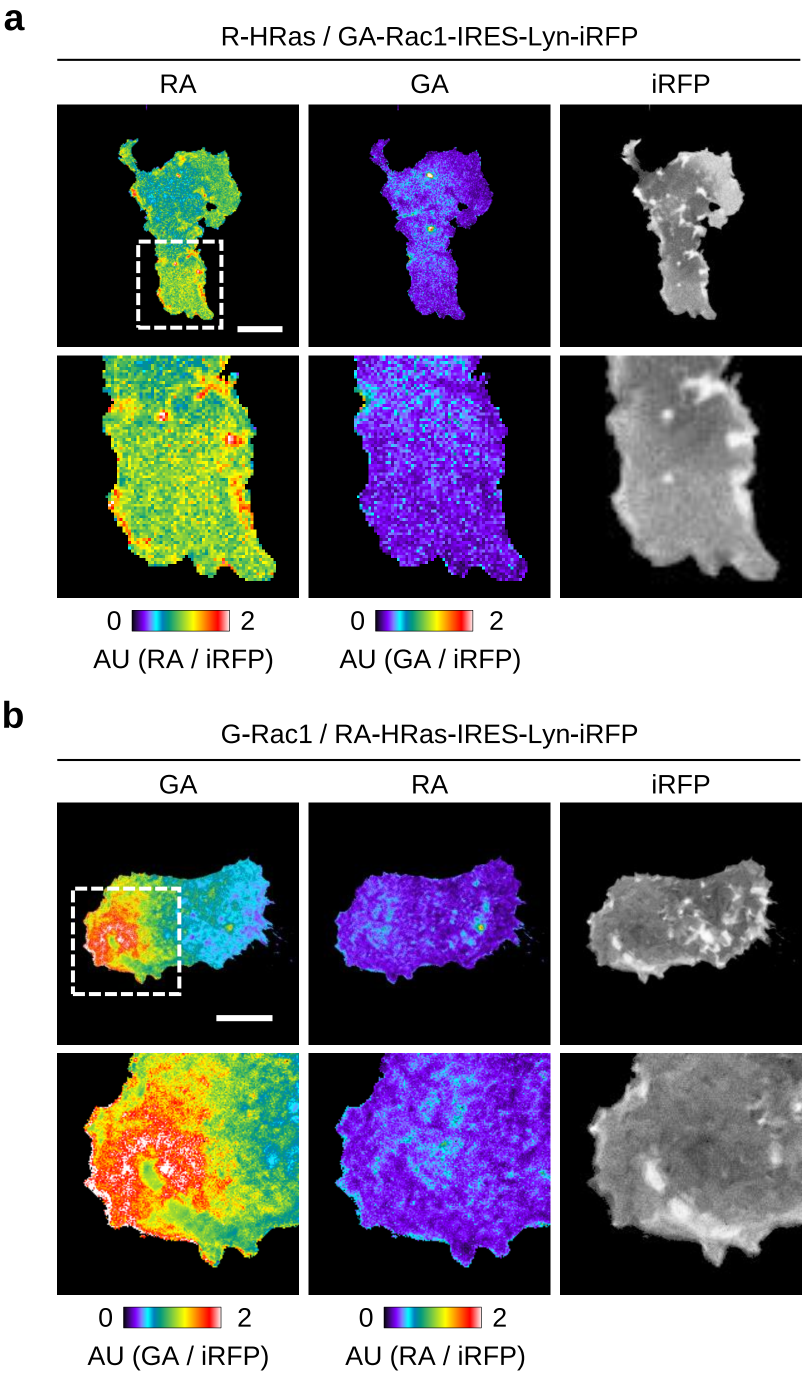

Supplementary Figure 8. Examination of cross-reactivity between RA-HRas and GA-Rac1 in a single cell. (a) Fluorescence images showing RA signals from R-HRas sensor and GA signals from GA-Rac1-IRES-Lyn-iRFP (without B-CRIB$_{Pak1}$) in a randomly migrating MDA-MB-231 cell. **(b)** GA signals from G-Rac1 sensor and RA signals from RA-HRas-IRES-Lyn-iRFP (without B-RBD$_{Raf1}$) in a randomly migrating MDA-MB-231 cell. Both GA and RA signals were normalized by iRFP signal. Intensity of Lyn-iRFP was also used to estimate expression level of GA-Rac1 or RA-HRas. Magnified images (bottom line) in **a** and **b** represents polarized areas of the cells marked with white dotted boxes. AU: arbitrary unit; Scale bars, 20 μm.

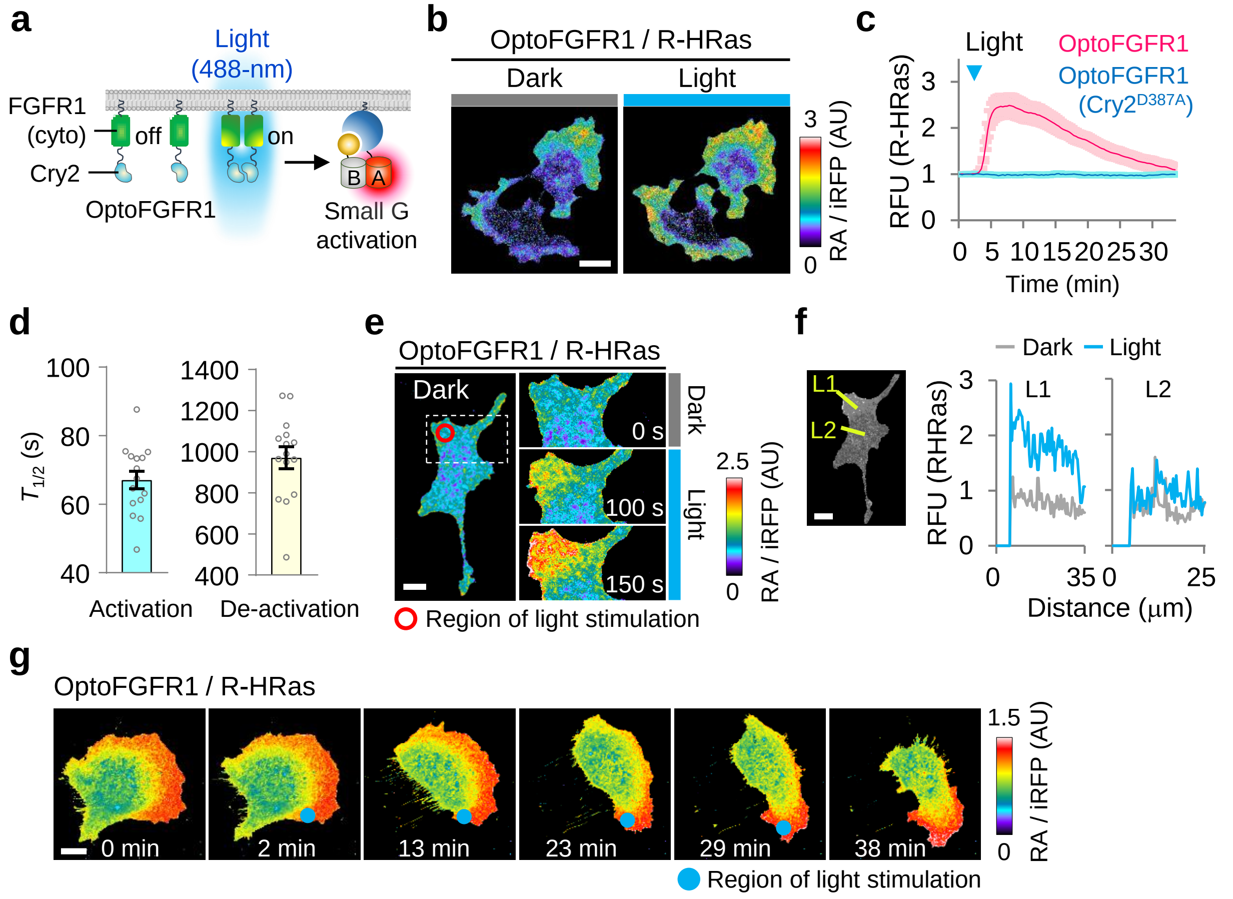

Supplementary Figure 9. Visualization of reversible and local changes of Ras activity in MDA-MB-231 cells by blue light-mediated OptoFGFR1 activation.

(a) Schematic of Ras activation by blue light-mediated optoFGFR1 activation. **(b)** Fluorescence images of MDA-MB-231 cells showing Ras activation upon whole-cell activation of optoFGFR1 by light. **(c)** Time-lapse measurement of relative fluorescence changes R-HRas sensor. OptoFGFR1(Cry2^{D387A}) is insensitive to light. n = 18 (magenta), 15 (gray). Blue light was delivered at t = 3 min. **(d)** Graph showing activation and deactivation kinetics of R-HRas measured from graph in **c**. **(e)** Visualization of local activation of HRas by OptoFGFR1. Light stimulation was applied to the peripheral region (indicated by red circle) of an MDA-MB-231 cell co-expressing R-HRas and optoFGFR1. Images showing local increase of Ras activity and membrane protrusion. **(f)** Activity changes of HRas in stimulated (L1) and non-stimulated regions (L2) by light. **(g)** Fluorescence images showing activity changes of Ras during light-directed cell migration. OptoFGFR1 was activated by repeated pulses of light delivered to peripheral region (blue circle) of an MDA-MB-231 cell co-expressing R-HRas and optoFGFR1. Lyn-iRFP was used in **b**, **e**, and **g** for normalizing the local fluorescence change caused by membrane fluctuation. AU: arbitrary unit; RFU: relative fluorescence unit; Error bars, s.e.m.; scale bars, 20 μm.

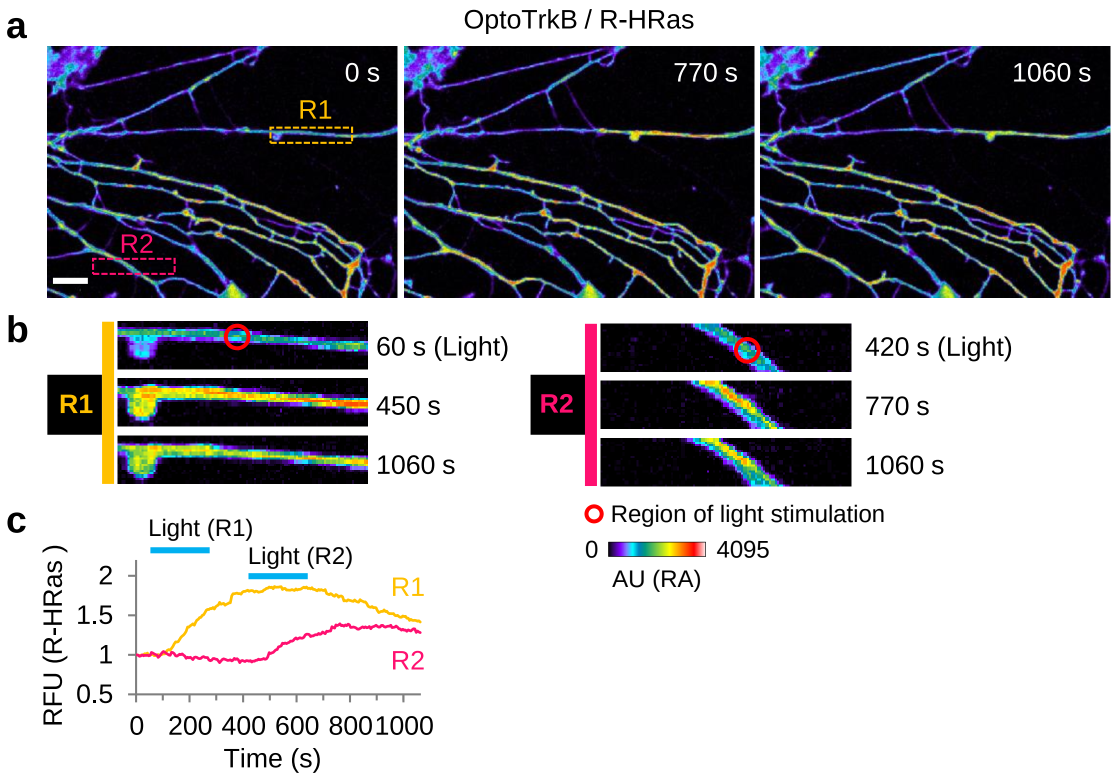

Supplementary Figure 10. Visualization of reversible and local changes of Ras activity in cultured hippocampal neurons by blue light-mediated OptoTrkB activation.

(a) Fluorescence Images of cultured rat hippocampal neuron (DIV-12) showing local increase of HRas activity visualized by R-HRas upon optoTrkB activation. Blue light was locally and sequentially delivered to two dendrites (red circles in R1 and R2 in **b**). The first region in R1 was stimulated at t = 60 s and the second region was stimulated at t = 420 s. **(b)** Enlarged fluorescence images of R1 and R2 in **a**, showing local and reversible changes of HRas. **(c)** Time-lapse graph showing changes of R-HRas intensity in R1 and R2, indicated as corresponding colors. Images were captured at 5-s intervals. AU: arbitrary unit; RFU: relative fluorescence unit.

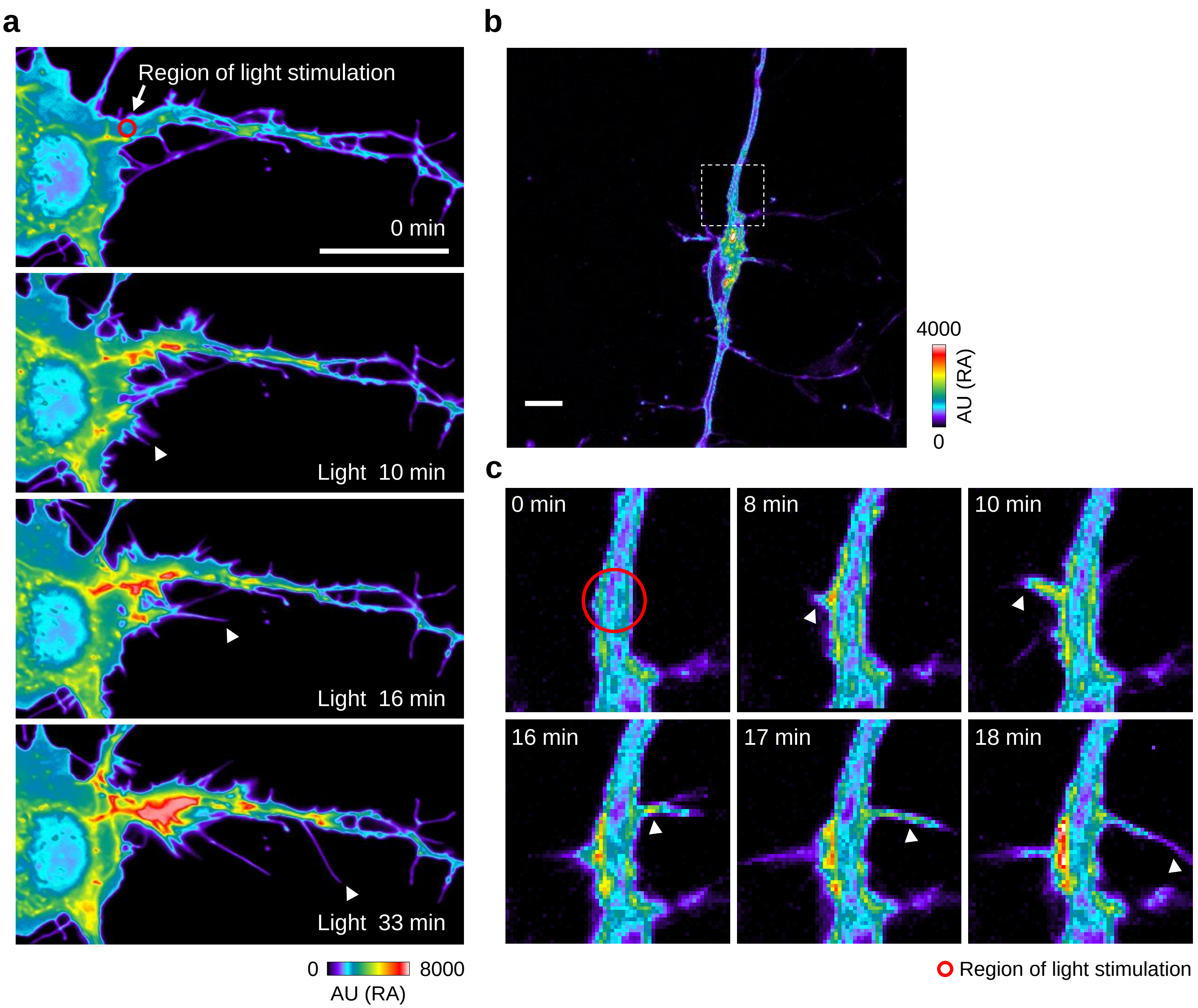

Supplementary Figure 11. Visualization of Ras activity and morphological changes of cultured hippocampal neurons under local activation of OptoTrkB.

(a) Fluorescence images of a cultured rat hippocampal neuron (DIV-10) showing local increase of R-HRas signal and morphological changes under activation of OptoTrkB at peripheral region of cell body. White arrowheads indicate filopodia-like membrane protrusion. Images were captured at 20-sec intervals. Blue light was locally and repeatedly delivered within the red circle for 15-min. **(b)** A fluorescence image of a cultured rat hippocampal neuron (DIV-10) expressing optoTrkB and R-HRas **(c)** Enlarged time-lapse images in **b** (white dotted box) showing local increase of R-HRas intensity and filopodia-like membrane protrusion (white arrowheads) upon light-triggered optoTrkB activation. Blue light was locally and repeatedly delivered within the red circle for 15-min. Images were captured at 20-sec intervals. AU: arbitrary unit; All scale bars, 20 μm

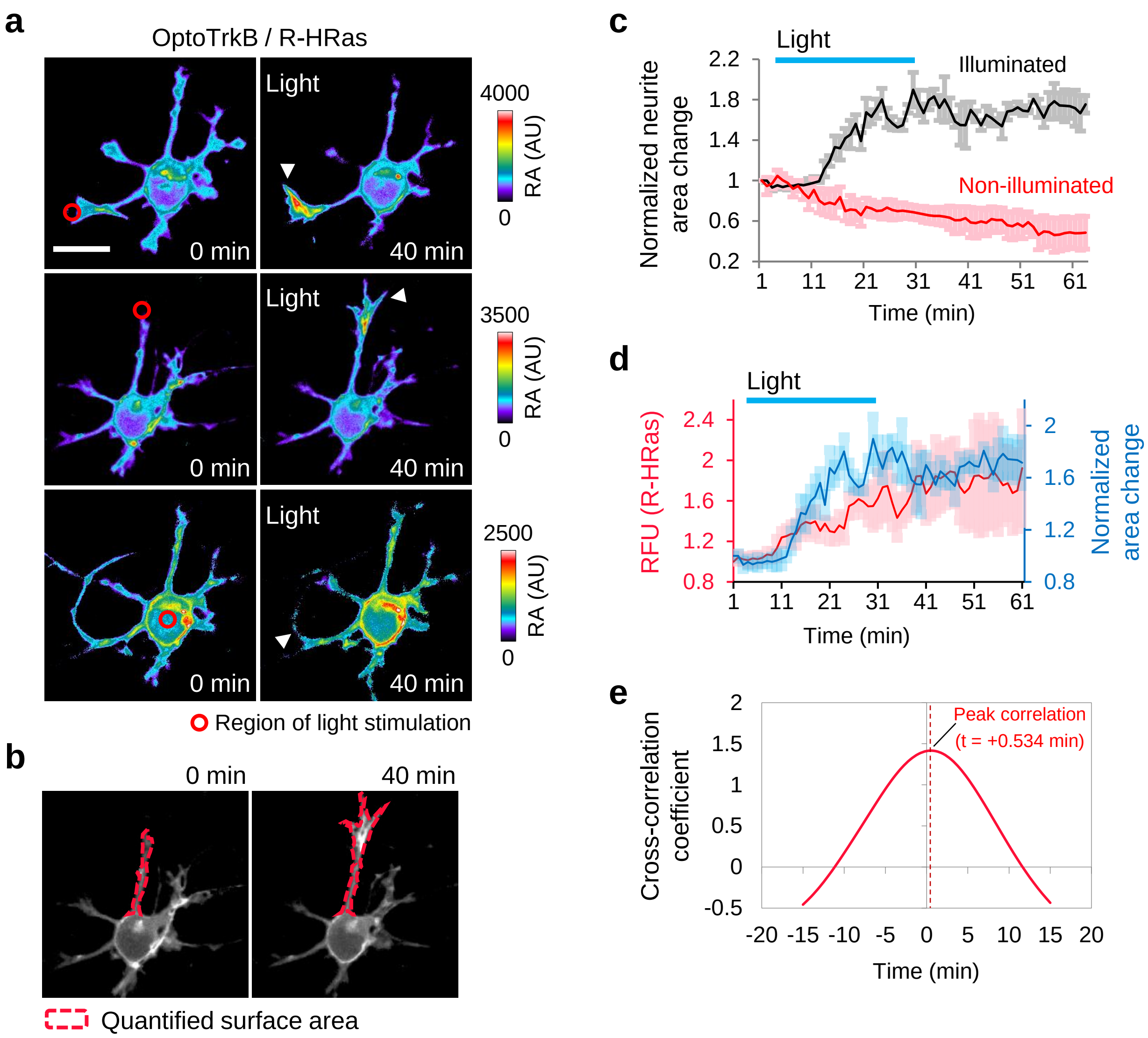

Supplementary Figure 12. Monitoring of Ras activity and neurite morphology in a cultured hippocampal neuron under local activation of OptoTrkB.
(a) Fluorescence images of a cultured rat hippocampal neuron (DIV-2) showing spatial changes of Ras activation and neurite morphology upon local activation of optoTrkB. Red circles indicates regions of blue light stimulation for 30-min (top-middle: near neurite tip, bottom: center of cell body). White arrowheads indicate regional changes of morphology and R-Ras signal. Scale bars, 20 μm. **(b)** Monochrome images represent how the area change of neurite outgrowth was measured in each frame (red dotted lines). **(c)** Quantification of morphological changes upon local OptoTrkB-triggered neurite extension compared with non-illuminated neurites. Data represents responses from three different neurites of the neuron in **a.** Images were captured at 1-min intervals. **(d)** A time-lapse graph showing local increase of R-HRas intensity and area change of illuminated neurites in **a**. **(e)** A graph showing a cross-correlation between the changes of Ras activity and the size of neurites in **d,** analyzed within the first 30 frames (during the period of light stimulation). Positive time value indicates Ras activity precedes neurite outgrowth. AU: arbitrary unit; RFU: relative fluorescence unit. Error bars, s.e.m.

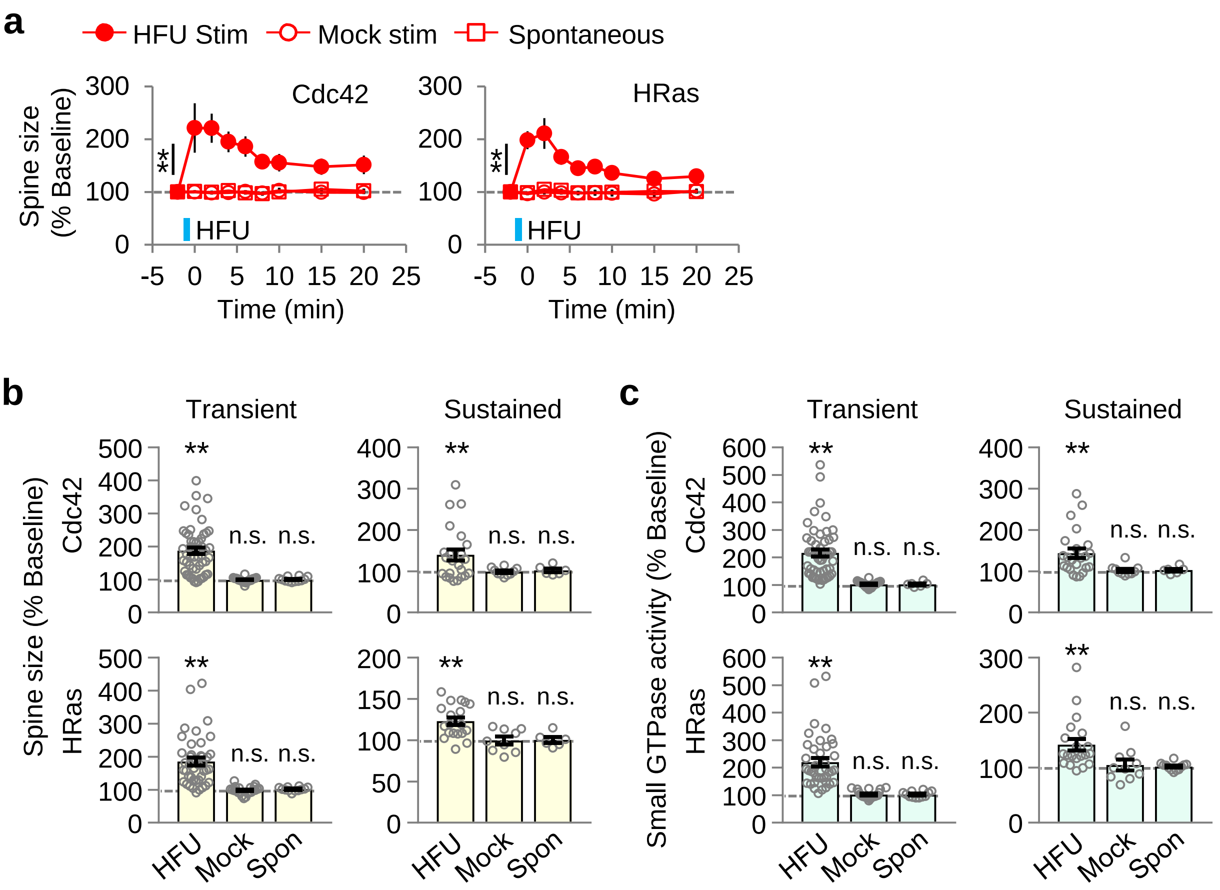

Supplementary Figure 13. Quantification of changes in spine volume and fluorescence intensity of G-Cdc42 and G-HRas in single dendritic spines undergoing structural plasticity. (a) HFU (high frequency uncaging) increased the volume of target spines compared with baseline in tdTomato and G-Cdc42 (left, filled circles; ** $P < 0.01$ at all post-HFU time points; $n = 26$ spines, 22 cells) and tdTomato and G-HRas (right, filled circles; ** $P < 0.01$ at all post-HFU time points; $n = 22$ spines, 19 cells) co-expressing layer 2/3 pyramidal neurons. In contrast, the volume of mock-stimulated (open circles, G-Cdc42: $n = 10$ spines, 10 cells; G-HRas: $n = 11$ spines, 11 cells) and unstimulated spines (open squares, G-Cdc42: $n = 23$ spines, 6 cells; G-HRas: $n = 23$ spines, 6 cells) did not increase. **(b)** HFU significantly increased the transient (left, changes over 0-2 min) and sustained (right, changes at 20 min) volume of target spines in both G-Cdc42 (top) and G-HRas (bottom) expressing layer 2/3 pyramidal neurons relative to mock-stimulated and unstimulated spines. **(c)** Transient (left) and sustained (right) green fluorescence intensities in target spines were significantly increased in both G-Cdc42 (top) and G-HRas (bottom) expressing layer 2/3 pyramidal neurons relative to mock-stimulated and unstimulated spines. ** $P < 0.01$. Statistical analysis were performed by Student's two-tailed t-test. Error bars: s.e.m.; n.s., not significant.

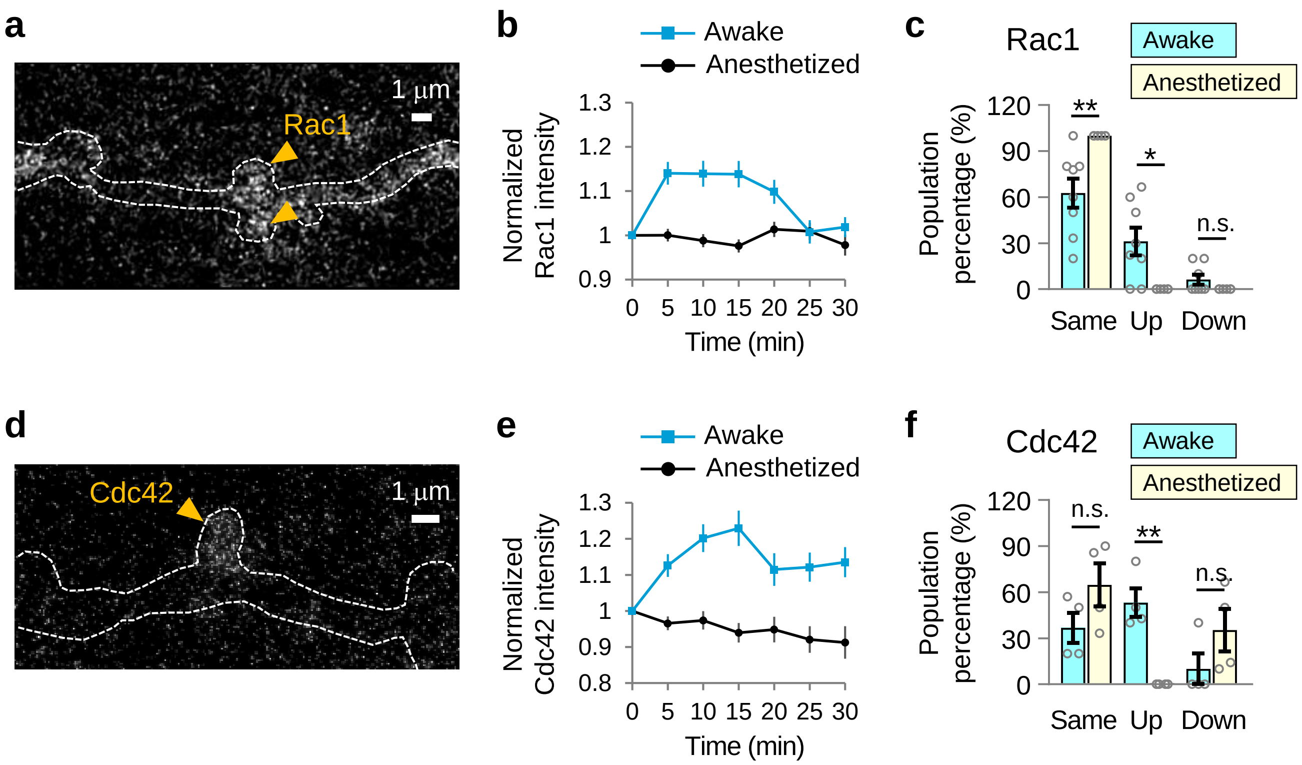

Supplementary Figure 14. Visualization of Rac1 and Cdc42 activity at the synapse resolution in awake behaving mice.

(a) A representative image of G-Rac1 fluorescence in a dendrite in awake state. Arrows indicate puncta exhibiting Rac1 signals. **(b)** A time-course graph showing Rac1 activity in awake and anesthetized states (n = 77 for the awake state, n = 52 for the anesthetized state, five mice). **(c)** Categorized Rac1 responses at 15 mins: same (>80% and <120%), up (≥120%), and down (≤80%). **(d)** A representative image of G-Cdc42 fluorescence in a dendrite in awake state. An arrow indicates punctum exhibiting Cdc42 signal. **(e)** A time-course graph showing Cdc42 activity in awake and anesthetized state (n = 26 for the awake state, n = 24 for the anesthetized state, four mice). **(f)** Categorized Cdc42 responses at 15 mins. *P < 0.05, **P < 0.01 by Student's two-tailed t-test, n.s., not significant. Error bars, s.e.m.
