# Supplementary material for: Intensiometric biosensors visualize the activity of multiple small GTPases in vivo
Source: Nat Commun. 2019 Jan 14;10:211. doi: 10.1038/s41467-018-08217-3 (PMC6331645; doi:10.1038/s41467-018-08217-3)
Supplement: Supplementary file 3 — Description of Additional Supplementary Files [file 41467_2018_8217_MOESM3_ESM.docx]

**Description of Additional Supplementary Files**

File Name: Supplementary Movie 1

Description: Visualization of reversible activation of Ras upon EGF and gefitinib treatment. Fluorescence images showing Ras activity during sequential treatment of EGF (50 ng ml-1 ) and EGFR inhibitor, gefitinib (400 nM). GA-KRas and B-RBDRaf1 were co-expressed in a HeLa cell by generating a P2A-based bicistronic expression vector (G-KRas). Images were captured at 30-s intervals for 30 min. Frame rate: 10 s/frame.

File Name: Supplementary Movie 2

Description: Dual-color visualization of KRas activity upon EGF treatment. Fluorescence images showing Ras activation upon treatment of EGF (50 ng ml-1 ). HeLa cells expressing G-KRas or R-KRas were co-cultured and simultaneously monitored upon EGF treatment. Images were captured at 20-s intervals for 19 min and 20 sec. Frame rate: 20 s/frame.

File Name: Supplementary Movie 3

Description: Simultaneous visualization of Rac1 and Ras activities in a single cell during random migration. Fluorescence ratio images representing simultaneous monitoring of Ras and Rac1 activities during migration of an MDA-MB-231 cell. Lyn-miRFP was co-transfected as a volume marker. Images were captured at 15-s intervals for 25 min and 45 sec. Frame rate: 30 s/frame.

File Name: Supplementary Movie 4

Description: Spatiotemporal Ras activation and directional cell migration using blue light-mediated OptoFGFR1 activation. Fluorescence images showing directional cell migration and corresponding Ras activation by locally induced OptoFGFR1 activation. Blue light was repeatedly applied to the peripheral region (indicated by blue circle) of the MDA-MB-231 cell migrating on the fibronectincoated plate. Lyn-iRFP was co-transfected as a volume marker. Images were captured at 20-s intervals for 38 min. Frame rate: 20 s/frame.

File Name: Supplementary Movie 5

Description: Visualization of local and reversible Ras activation in dendrites of cultured hippocampal neuron under blue light-meidated OptoTrkB activation. Images showing local increase of R-HRas intensity upon OptoTrkB activation. Blue light was locally and sequentially delivered to three dendrites of hippocampal neuron (DIV-12) indicated by white arrows. Images were captured at 5-s intervals for 17 min and 40 sec. Frame rate: 30 s/frame.
